# An Analysis of Individual Differences in Recognizing Monosyllabic Words Under the Speech Intelligibility Index Framework

**DOI:** 10.1177/2331216518761773

**Published:** 2018-03-13

**Authors:** Yi Shen, Allison B. Kern

**Affiliations:** 1 Department of Speech and Hearing Sciences, Indiana University Bloomington, Bloomington, IN, USA

**Keywords:** speech understanding, articulation index, relative weights, masking

## Abstract

Individual differences in the recognition of monosyllabic words, either in isolation (NU6 test) or in sentence context (SPIN test), were investigated under the theoretical framework of the speech intelligibility index (SII). An adaptive psychophysical procedure, namely the quick-band-importance-function procedure, was developed to enable the fitting of the SII model to individual listeners. Using this procedure, the band importance function (i.e., the relative weights of speech information across the spectrum) and the link function relating the SII to recognition scores can be simultaneously estimated while requiring only 200 to 300 trials of testing. Octave-frequency band importance functions and link functions were estimated separately for NU6 and SPIN materials from 30 normal-hearing listeners who were naïve to speech recognition experiments. For each type of speech material, considerable individual differences in the spectral weights were observed in some but not all frequency regions. At frequencies where the greatest intersubject variability was found, the spectral weights were correlated between the two speech materials, suggesting that the variability in spectral weights reflected listener-originated factors.

## Introduction

The recognition of speech in a noisy background is a comprehensive process that involves peripheral, central auditory, and cognitive factors (e.g., Committee on Hearing and Bioacoustics and Biomechanics, 1988; Humes et al., 2012). Although audibility is important for understanding speech, individual listeners exhibit differences in speech recognition performance even when audibility is controlled for (e.g., Humes, Kidd, & Lentz, 2013). The current study investigates the possibility of analyzing individual differences in speech recognition using the Speech Intelligibility Index (SII, American National Standards Institute [ANSI], 1997) model.

The SII reflects the proportion of total speech information available to the listener. It is given by:

(1) $$\text{SII}=\sum_{i}w_{i}A_{i}$$

where *w_i_* is the band importance function, which represents the relative importance of speech information, or the spectral weight, for the *i*th frequency band. *A_i_* is the band audibility function, which represents the audibility of speech signal in the *i*th frequency band. The spectral weights across all frequency bands sum to 1, and the value of *A_i_* ranges between 0 and 1. Therefore, the SII is constrained to be between 0 and 1. Previous studies have demonstrated that the SII is capable of predicting average speech-recognition scores in quiet and in steady-state noise for a variety of test materials, including nonsense syllables (French & Steinburg, 1947; Pavlovic & Studebaker, 1984), monosyllabic words (e.g., ANSI S3.5-1969; Black, 1959; Duggirala, Studebaker, Pavlovic, & Sherbecoe, 1988; Tillman & Carhart, 1966), spondees (Dubno, Dirks, & Morgan, 1984), and sentences (Grant & Braida, 1991; Healy, Yoho, & Apoux, 2013; Kalikow, Stevens, & Elliott, 1977; SPIN; Studebaker, Pavlovic, & Sherbecoe, 1987; Studebaker, Sherbecoe, & Gilmore, 1993). The core assumption underlying the SII is that audibility in various frequency regions contributes to speech understanding differentially according to the band importance function. The band importance function is a function relating spectral weights to frequency. If a certain frequency region exhibits a high weight, then it means that this frequency region is relatively more essential for speech intelligibility. Otherwise, if a frequency region has a low weight, then this frequency region carries less importance.

Although the SII model is predictive of average speech recognition performance, it is known that homogeneous performance would not be expected across listeners, especially hearing-impaired listeners. In particular, a number of studies have suggested large variability in the benefits from high-frequency (i.e., >3 kHz) speech audibility (e.g., Amos & Humes, 2007; Ching, Dillon, & Byrne, 1998; Hogan & Turner, 1998; Horwitz et al., 2008). For some hearing-impaired listeners, high-frequency amplification can even lead to performance degradation (e.g., Amos & Humes, 2007). Ching et al. (1998) systematically explored the approaches of extending the SII model to improve its predictive power to individual data. These authors suggested that a proportion of individual differences in speech recognition may be modeled using a multiplicative, frequency-dependent proficiency factor fitted for each individual listener (i.e., Equation (9) in Ching et al., 1998). That is,

(2) $${\text{SII}}_{m}=\sum_{i}w_{i}A_{i,m}L_{i}P_{i,m}$$

where SII*_m_* is the SII for the *m*th individual listener; *w_i_* is the band importance function; and *A_i,__m_* is the band audibility function, which represents the audibility of speech signal in the *i*th frequency band according to the *m*th listener’s audiogram and the relative intensity between the target speech and masker (i.e., Target-to-Masker ratio or TMR). *L_i_* is a level-dependent distortion factor to account for the phenomenon that speech understanding degrades at high speech levels. *P_i,__m_* is the proficiency factors for the *i*th band and *m*th listener. As an alternative to the formulation of Ching et al. (1998), one can treat *w_i_*, *L_i_*, and *P_i,__m_* as components of an individualized band importance function *w′_i,__m_*:

(3) $${\text{SII}}_{m}=\sum_{i}w{'}_{i,m}A_{i,m}$$

This individualized band importance function reflects suprathreshold factors related to the type of speech material, speech level, and listening proficiency or strategy.

To apply the individualized version of the SII model to predict variations in speech understanding across listeners for various types of speech materials, an appropriate approach to estimate the band importance function and fit the SII model to individual listeners’ speech-recognition performance is required. The classic approach for estimating the band important function (e.g., French & Steinberg, 1947) involves the following steps. First, speech recognition is measured at high signal-to-noise ratio under both low-pass and high-pass conditions. As the cutoff frequency of the low-pass filter increases, the performance increases due to the availability of more frequency bands. On the other hand, as the cutoff frequency of the high-pass filter increases, the performance decreases. At a certain intermediate cutoff frequency, equal performance is achieved for the low- and high-pass conditions, which can be considered as the performance associated with half of the speech information (i.e., an SII of 0.5). Repeating the aforementioned procedure provides a function relating the SII to speech-recognition performance, that is, a link function. Second, using the obtained link function, the relationship between performance and cutoff frequency is converted into a function that describes how the SII accumulates (for the low-pass condition) or dissipates (for the high-pass condition) as the cutoff frequency increases. Third, differentiating the aforementioned function and then averaging across the low- and high-pass conditions lead to the final estimate of the band importance function.

Calandruccio and Doherty (2007) pointed out that the low- and high-pass filtered speech used to derive the band importance function does not represent realistic listening scenarios. In an effort to probe spectral weights while utilizing relatively less artificial stimuli, Calandruccio and Doherty (2007) adopted a correlational method that obtained spectral weights based on the correlation between the signal-to-noise ratio within each frequency band and the correctness in recognizing keywords in sentences. These authors showed that the spectral weights obtained using the correlational method deviated from those expected using low- and high-pass stimuli. There were also noticeable differences between spectral weights for nonsense syllables and sentences. Apoux and Healy (2012) developed another technique, namely the *compound* technique, to obtain estimates of the band importance function while addressing issues associated with the classic approach of high-pass and low-pass filtering speech materials. This technique assessed the importance of each frequency band in turn. For each target band, the speech stimulus was presented through the target band and four other frequency bands drawn at random on a trial-by-trial basis. These authors reevaluated the band importance functions reported for the W22 words (Hirsh et al., 1952) and the SPIN sentences (Kalikow et al., 1977) using the compound technique and found that the band importance functions differed substantially depending on the method used (Healy et al., 2013).

Bosen and Chatterjee (2016) were among the first to estimate band importance functions from individual listeners. These authors filtered speech into 20 frequency bands and selected a subset of the bands (7 out of 20 bands) to be presented on each trial. The selection of the presentation bands was governed by rules to ensure that (a) the selected bands spread across a wide frequency range; (b) each band occurred equal number of times within an experimental run; and (c) the co-occurrences of pairs of bands were balanced. Using noise-vocoded sentences, the band importance functions estimated from normal-hearing listeners were qualitatively similar to those reported for speech presented in quiet by Healy et al. (2013).

Based on previous research efforts, the individualized band importance function provides a framework to investigate the source of individual differences in speech recognition. With an individually fitted SII model, it is possible to assess whether individual differences are mainly governed by audibility, spectral weights, or general speech-recognition proficiency. Therefore, it may be beneficial to develop a relatively rapid experimental procedure for routine estimation of the individualized band importance function. For this purpose, the aforementioned approaches to evaluate spectral weights are limited in that they typically require a time-consuming process for data collection. In some cases, due to the limited number of unique tokens in the speech corpuses, each estimate of the spectral weights requires combining data from multiple listeners to prevent repeating tokens for each listener. For the classic approach, for example, Studebaker et al. (1993) obtained their estimates of the band importance function using 6,400 experimental trials pooled across 12 listeners. The correlational method used by Calandruccio and Doherty (2007) required 600 sentences (3,000 keywords) to obtain weight estimates, which took approximately 3 hours. Each of the band importance functions reported by Healy et al. (2013) using the compound technique (Apoux & Healy, 2012) were based on 1,176 trials pooled over three groups of listeners, which took approximately 6 hours. The laborious process involved in the estimation of the spectral weights prevents conducting the aforementioned approaches to be on a routine basis.

In the current study, a new adaptive procedure, namely the quick-band-importance-function (qBIF) procedure, is used similar to the approaches used by Calandruccio and Doherty (2007), Healy et al. (2013), and Bosen and Chatterjee (2016) with modifications to shorten the required testing time. One of these modifications is to limit the resolution of the band importance function so that it only includes six octave bands. This compromise in spectral resolution allows the weight estimates to converge more rapidly since only a small number of free parameters are estimated. Another modification is that the qBIF procedure uses a Bayesian adaptive algorithm to iteratively optimize the stimulus for each test trial. This algorithm omits stimulus configurations that are not informative to the spectral weights, which aims to improve the test efficiency. In the following, the qBIF procedure will be first described in detail, then an initial feasibility study using the qBIF procedure to analyze individual differences in speech recognition performance will be presented. In the feasibility study, the parameters for the SII model were estimated using two different types of speech materials. Whether the variability in the parameter estimates is merely random measurement errors or they reflect factors inherent to individual listeners was investigated. It will be shown that correlations were found between the two sets of parameter estimates using two different speech materials. The observed within-subject consistency demonstrates the potential for characterizing individual differences for speech recognition in noise using the parameters of the SII model.

## The QBIF Procedure

### Rationale

The qBIF procedure is similar to previous approaches to estimate the band importance function using the compound technique (e.g., Bosen and Chatterjee, 2016; Healy et al., 2013) with a few modifications. First, the qBIF procedure varies not only the frequency bands to be presented but also the TMR on a trial-by-trial basis. Instead of randomly running through a predetermined stimulus set, the qBIF procedure optimizes the stimulus choice iteratively based on the previously collected responses on the same qBIF run. The optimization algorithm maximizes the expected information gain for each trial, which leads to two potential benefits. First, it may improve the test efficiency by not sampling stimuli that are not informative to the estimated parameters of the SII model. Second, the optimization algorithm can adapt to individual listeners’ level of performance, so that the overall speech-recognition score after a qBIF run would be relatively consistent across listeners. On the other hand, if a predetermined stimulus set is used, it is possible that the stimuli selected may be too easy for some listeners while too difficult for others.

A second noticeable difference between the qBIF and many previous procedures is that the qBIF procedure estimates the band importance function with a small number of frequency bands (i.e., six octave bands). Consequently, the qBIF procedure is not able to estimate some of the fine variations in the spectral weights. The rationale for this compromise in spectral resolution is twofold. First, the reduced total number of free parameters means that a smaller number of experimental trials may be required to estimate the BIF. Second, as the number of frequency bands (*N*_band_) increases, the number of potential stimulus choices increases. When *N*_band_ is too large, the computational effort of the iterative stimulus optimization algorithm may be too high so that a significant delay is required between consecutive experimental trials. The effect of *N*_band_ will be explored further below using computational simulations.

### Implementation

**Figure 1.**
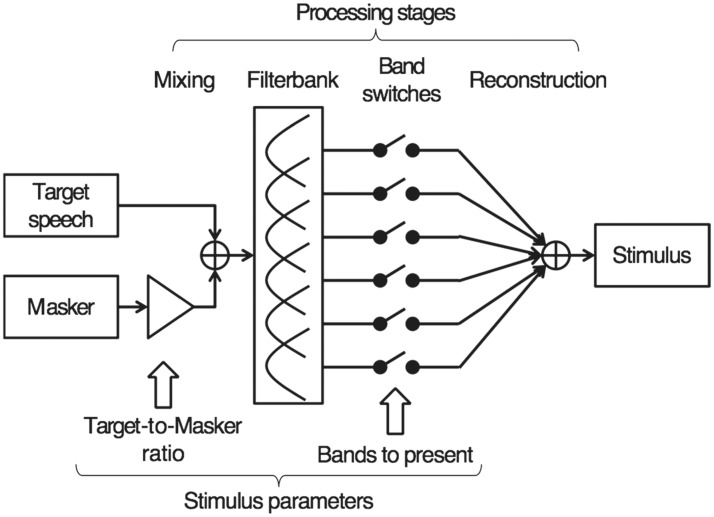
The schematic for the signal processing stages in the qBIF procedure.

During the qBIF procedure, the stimulus presented on each trial is optimized iteratively following each trial, which maximizes the expected information gain from the following trial. Before each trial, the stimulus optimization algorithm selects one stimulus from a pool of all possible stimuli. Figure 1 illustrates how the stimuli in the qBIF procedure are generated. The target speech and competing masker are first mixed together at the specified TMR and then the target-masker mixture is passed through a filter bank. The filter bank consists of six octave-frequency bands (i.e., *N*_band_ = 6), centered at 250, 500, 1000, 2000, 4000, and 8000 Hz. Each of the filters is constructed as 12th-order butterworth filters (using the MATLAB command *fdesign.octave* included in the DSP Systems Toolbox) with 36 dB/oct roll-off at each of its cutoff frequencies. The outputs from these filters are used to reconstruct the target-masker mixture, but with the signals from a subset of the frequency bands omitted during reconstruction. Therefore, the stimulus on a given trial is determined by the TMR and a switch vector **n** that governs which of the six octave bands that allows the target and masker to pass (i.e., *n_i_* = 0 or 1, *i* = 1,…,6). To construct the pool of possible stimuli, the possible TMR values are from −5 dB to 15 dB with 5-dB spacing; and the possible values for **n** is limited so that the number of bands included for speech presentation ranged from two to five bands. This leads to 56 possible values for **n** and 280 unique combinations of TMR and **n** in the pool of possible stimuli.

In the qBIF procedure, speech recognition performance is described by a logistic link function:

(4) $$p=[1+e^{-\beta (30\mathrm{SII}\text{-}15-\text{SRT})}{]}^{-1}$$

In Equation (4), *p* is the probability of correctly recognizing a keyword in speech; the speech recognition threshold (SRT) indicates the TMR corresponding to 50% correct recognition; *β* is the slope parameter of the link function with greater *β* values indicating steeper slopes; and the SII is given by Equation (3). At suprathreshold speech levels, the band audibility function *A_i_* is given by:

(5) Ai=TMRi'+1530

where TMR_i_′ is the TMR in the *i*th frequency band and bounded between −15 and 15 dB, such that 0 ≤ *A_i_* ≤ 1.

The current implementation of the SII model has a total of eight free parameters (spectral weights in the six octave bands, SRT, and *β*). These parameters can be estimated using logistic regression following each experimental trial of the qBIF procedure. In the following, the parameter estimates following the *k*th trial are written as a Vector **φ***_k_* in an eight-dimensional parameter space. The covariance matrix for **φ***_k_*, provided by the logistic regression, will be noted as **P***_k_*. Note that **φ***_k_* and **P***_k_* describe a multivariate posterior parameter distribution with **φ***_k_* being the mean of the distribution and **P***_k_* describing the covariance matrix of the distribution. Assuming that the distribution takes the form of a multivariate Gaussian distribution, the spread of the posterior parameter distribution can be quantified as the entropy, which is proportional to the determinant of the covariance matrix **P***_k_*.

Following the *k*th trial, the qBIF procedure optimizes the stimulus choice within the pool of possible stimuli. The optimization algorithm is based on an entropy-based criterion, such that the expected entropy for the posterior parameter distribution following the *k* + 1th trial would be minimized (see also Kontsevich & Tyler, 1999; Lesmes, Lu, Baek, & Albright, 2010; Shen & Richards, 2013a, 2013b; Shen, Sivakumar, & Richards, 2014)

(6) $$\{\mathrm{TMR},n\}={\arg\min}_{{\mathrm{TMR}}^{'},n^{'}}\{E[\text{ln}|P_{k+1}^{'}|]\}$$

where |**P***_k_*_+1′_| is the determinant of the covariance matrix for the parameter distribution following the *k* + 1th trial with the hypothetical stimulus specified by TMR′ and **n**′; and *E*(.) indicates the expected value across the two possible responses (i.e., correct or incorrect) collected from the *k* + 1th trial.

In practice, the performance of the aforementioned one-step-ahead search algorithm (Equation (6)) depends on the accuracy of the interim parameter estimate **φ***_k_*. However, at the beginning of data collection, there may not be sufficient data to establish a reasonable interim estimate and to direct the stimulus sampling in a meaningful manner. To address this issue, the one-step-ahead search algorithm is not activated until some initial training data is collected. Here, *training* refers to the training of the qBIF algorithm, not the listener. The initial TMR is 15 dB and only one frequency band is omitted. Therefore, during the first six trials, each of the six bands is omitted once in random sequence, followed by 15 trials with pairs of bands omitted, also in random sequence. This procedure is repeated for combinations of three, and then four, omitted bands until (a) the number of training trials is greater than 10 and the performance score drops below 65% or (b) the number of training trials is greater than 50. These early trials are sequenced so that each qBIF run always starts from relatively easy conditions (a 15-dB TMR with only one band omitted), and the training data set contains sufficient incorrect responses to enable a meaningful logistic regression. The one-step-ahead search algorithm (Equation (6)) is activated once the collection of the training data is complete.

### Simulations

To validate the implementation of the qBIF procedure, as described earlier, and to assess its efficiency, computational simulations were conducted. In these simulations, simulated listeners were constructed using the standard SII model and the band importance functions were estimated from the simulated listeners. The simulations focused on: (a) the effect of the total number of frequency band *N*_band_ on the accuracy and computational efforts of the qBIF procedure and (b) the efficiency of the one-step-ahead search algorithm (Equation (6)) as compared with other stimulus sampling strategies.

The simulated listeners produced responses, in terms correct or incorrect keyword recognition, according to a link function:

(7) $$p=(1-10^{-\mathrm{SII}P/Q}{)}^{N}$$

where *P*, *Q*, and *N* are the three free parameters of the link function (e.g., Dirks, Bell, Rossman, & Kincaid, 1986; Fletcher & Galt, 1950; Studebaker & Sherbecoe, 1991; Kates, 2013; Jin, Kates, Lee, & Arehart, 2015; Jin, Kates, & Arehart, 2017). The link function for the simulated listeners (Equation (7)) was different from the logistic link function used for the qBIF procedure (Equation (4)). This was implemented to verify that the qBIF procedure was relative robust against specific formulations of the link function. The SII in Equation (7) was calculated based on the stimulus parameters TMR and **n** for each simulated trial. For each simulated listener, *P* was set to 1 and all other model parameters (spectral weight in each of the *N*_band_ frequency bands, *Q*, and *N*) were randomly determined. The spectral weights were constructed by first randomly drawing *N*_band_ candidate values from a uniform distribution spanning 0 and 1 and then normalizing the drawn values so that they summed to 1. The value for *Q* was randomly drawn from a uniform distribution spanning 0.2 and 0.5. The value for *N* was randomly drawn from a uniform distribution spanning 2 and 12.

For the effect of *N*_band_, four values of *N*_band_, 4, 6, 8, and 10, were tested. For each of the *N*_band_ values, 100 simulated listeners were constructed. For each simulated listener, the band importance function was estimated using a qBIF run containing 500 simulated trials. The accuracy of the qBIF procedure was quantified as the root-mean-squared (RMS) deviation from the estimated spectral weights to the true weights, that is, the RMS error. The RMS errors were normalized so that they reflected the error within an octave frequency range. This normalization procedure was used to counter balance the fact that as *N*_band_ increases the spectral weights decreases on average because they sum to 1. Therefore, the expected RMS errors without normalization are expected to be smaller for higher values of *N*_band_.

**Figure 2.**
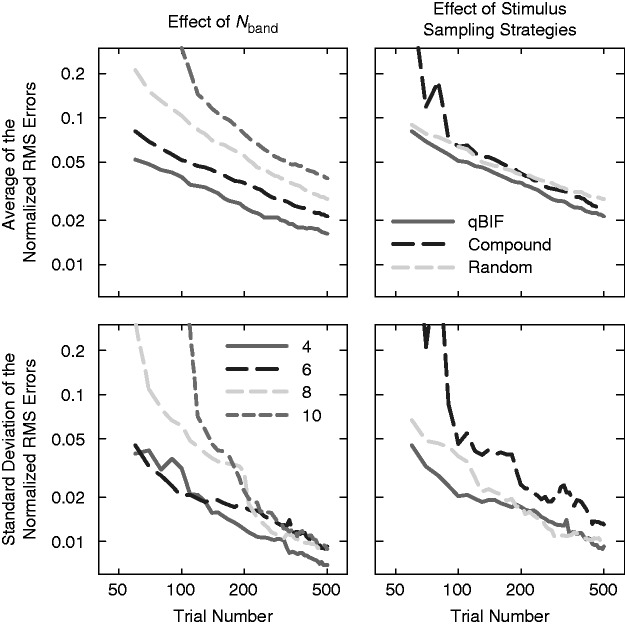
The mean (top panels) and standard deviation (bottom panels) of the normalized RMS errors across 100 simulated listeners as functions of trial number. In the left panels, results are plotted for the qBIF procedures implemented with the total number of frequency bands being 4, 6, 8, and 10 bands (different line styles). In the right panels, results are plotted for three stimulus sampling strategies (different line styles). RMS = root-mean-squared; qBIF: quick-band-importance-function; quick-band-importance-function.

The upper left panel of Figure 2 plots the normalized RMS error, averaged across the 100 simulated listeners, as a function of the number of trials for the four values of *N*_band_. As the number of trials increases, the normalized RMS error decreases. As *N*_band_ increases, the normalized RMS error increases; therefore, the accuracy of the band importance function estimated using the qBIF procedure becomes poorer for a larger number of implemented frequency bands. The lower left panel of Figure 2 plots the standard deviation of the normalized RMS errors across simulated listeners. As the trial number increases, the standard deviation decreases, indicating that the qBIF procedure is more robust to listener variations as more trials of data are collected. Below 250 trials, the standard deviation tends to be larger for higher values of *N*_band_, suggesting that the robustness of the qBIF procedure is greater for smaller values of *N*_band_. Above 250 trials, the standard deviation becomes similar across *N*_band_.

The simulations also showed that as *N*_band_ increases, the qBIF procedure required greater computational resources. For 4, 6, 8, and 10 bands, the average computing time for the one-step-ahead search algorithm running at a processor speech of 3.60 GHz were 0.16, 0.75, 3.62, and 8.73 s per trial, respectively. This means that for standard personal computers, the qBIF procedure may require a considerable amount of computing time between consecutive trials for a large *N*_band_. As stated earlier, one of the reasons that the qBIF procedure was implemented with six octave bands in the current study was to prevent the computational delay from undermining the efficiency of data collection.

For the effect of stimulus sampling strategies, the number of frequency bands *N*_band_ was fixed to be six. Besides the one-step-ahead search algorithm in the qBIF procedure, two other strategies were considered, namely the *Compound* strategy and the *Random* strategy. The Compound strategy was inspired by the compound technique developed by Apoux and Healy (2012). The Compound strategy identifies one of the six frequency bands as the target band. For each pair of trials, two bands other than the target band were randomly drawn and were used to present the speech on both of the trials. The two trials in the pair were different in that the target band was included to present the speech in one trial while it was not included for stimulus presentation on the other trial. For each target band, the Compound procedure ran through all 10 possible band combinations (i.e., 20 trials) in random order before a new target band was identified. Each of the six frequency bands was served as the target band one at a time (from band 1 to band 6 in that order), and this process was repeated until a total of 500 trials were reached. For the Compound strategy, speech was only presented to the simulated listeners in quiet.

The Random strategy was akin to the procedure developed by Bosen and Chatterjee (2016). It presented speech in a subset of the six bands, and the number of bands included for speech presentation was from 2 to 5. This means that there were a total of 56 possible band combinations. These 56 combinations were tested in random order and repeated until a total of 500 trials were reached. As for the Compound strategy, only speech-in-quiet was presented to the simulated listeners. For each of the three stimulus sampling strategies, 100 simulated listeners were constructed. The band importance functions for all simulated listeners were estimated using logistic regression, and the accuracy of the estimates was quantified using the RMS error as before.

The upper right panel of Figure 2 plots the normalized RMS error, averaged across the 100 simulated listeners, as a function of the number of trials for the three stimulus sampling strategies. As the number of trials increases, the normalized RMS error decreases for all three strategies. Above approximately 100 trials, all three strategies leads to similar RMS errors, with the qBIF procedure performs slightly better than the other two. For example, to reach a normalized RMS error of 0.05, about 113 trials are required for the qBIF procedure, 130 trials are required for the Random strategy, and 160 trials are required for the Compound strategy on average. For trial numbers less than 100, the accuracy of the Compound strategy was poorer than the other two strategies, which may be because that the Compound strategy ran through target bands in a sequential fashion. The lower right panel of Figure 2 plots the standard deviation of the normalized RMS errors across simulated listeners. The standard deviation decreases as the trial number increases, indicating that the robustness against variations among simulated listeners improves with a greater number of trials for all three stimulus sampling strategies. The standard deviation of the normalized RMS errors is the highest for the Compound strategy. Below 200 trials, the standard deviation is lower for the qBIF procedure than the Random strategy, while above 200 trials, these two strategies lead to similar standard deviations.

The recognition score across the 500 simulated trials for each of the three stimulus sampling strategy reveals whether the most informative stimuli were selected by the strategy. In principle, an efficient strategy should sample stimuli near a performance level of 50%, while sampling stimuli that lead to performance ceiling or floor would not be efficient. The simulated recognition scores averaged across the 100 simulated listeners were 53.7%, 53.1%, and 65.8% and the standard deviations were 5.1%, 15.1%, and 12.3% for the qBIF procedure, the Compound strategy, and the Random strategy, respectively. This suggests that the qBIF procedure concentrates stimuli consistently near 50% correct, while for the other two strategies, the recognition scores depend on individual simulated listeners since these two strategies do not adapt to individual listeners’ levels of speech-recognition proficiency.

Overall, the simulations presented earlier show that the qBIF procedure implemented with six octave bands can provide efficient estimation of the band importance function with satisfactory robustness against listener variations. Below, results will be presented for the band importance functions estimated from a group of normal-hearing listeners using the qBIF procedure.

## Methods

### Listeners

Thirty listeners (16 females and 14 males) were recruited for the current study, all of whom were native speaker of English but naïve to speech recognition experiments. The listeners were between the ages of 18 and 26 years. All listeners had audiometric thresholds at or lower than 15 dB HL between 250 and 8000 Hz in both ears. The ear with lower pure-tone average thresholds, that is, the average threshold across 500, 1000, and 2000 Hz, was chosen as the test ear. In cases where the PTA thresholds from the two ears were identical, the left ear was used as the test ear. The experimental protocol was approved by the institutional review board at Indiana University. Informed consent was obtained from all listeners.

### Stimuli

The target speech stimuli included words from the NU6 list corpus (Studebaker et al., 1993) recorded by Auditec of St. Louis and the Revised SPIN sentence list recorded by Bilger, Nuetzel, Rabinowitz, and Rzeczkowski (1984) at the University of Illinois. Both lists were spoken by an adult male speaker with an American accent. The NU6 word list consisted of four lists of 50 test items each. Each test item contained a carrying phrase followed by a keyword (i.e., 50 keywords per list). Lists 1 through 3 were used in the current study. The SPIN sentences consisted of eight lists of 50 sentences. Each list contained 25 high-context (SPIN-High) and 25 low-context (SPIN-Low) sentences. The high-context sentences from Lists 1 through 8 were used in the current study. The masker presented was a multitalker babble from the original test materials. For the NU6 material, the babble noise consisted of 20 young adults (including both male and female talkers) who were recorded simultaneously reading different passages. For the SPIN material, the noise was a 12-talker babble, including both male and female talkers. The target speech and babble noise were combined using the procedure described in the previous section (also see Figure 1). The filtering of the stimuli was applied to the entire stimulus for each trial, including the carrying phrase for NU6 and the entire sentence for SPIN, not just to the keywords.

All stimuli were presented at a sampling rate of 44100 Hz. They were presented to the listeners via a 24-bit soundcard (Microbook II, Mark of the Unicorn, Inc.) and a headphone (HD280 Pro, Sennheiser electronic GmbH & Co. KG). During the experiment, the listeners were seated in a sound-attenuating booth.

### Procedures

Following audiometric threshold measurements, half of the listeners were tested using the NU6 lists first followed by the SPIN lists, while the other half began with the SPIN material. For NU6, the listener was presented with a sentence that consisted of a carrier phrase: “Say the word,” and a monosyllabic keyword. The listener’s task was to verbally repeat the keyword. The experimenter was seated outside of the sound-attenuating booth and monitored the listener’s verbal responses via a talk-back microphone, which was a part of a clinical audiometer (GSI Pello, Grason-Stadler, Inc.). The responses were scored in terms of correctness. When a response was not clear to the experimenter, the listener was instructed to repeat the response. The Lists 1 to 3 of the NU6 corpus were tested in random order, and repeated once in the same random order, leading to a total of 300 trials. For each of the NU6 lists, the order of the keywords was shuffled, so even though each list was repeated twice, the listener was not able to identify the keyword based on the memorized word order. During the 300 trials, the stimulus was adaptively modified on a trial-by-trial basis by the qBIF procedure. For SPIN, a sentence with high semantic context was presented on every trial and the listener was instructed to repeat the last word of the sentence. Lists 1 to 8 of the SPIN corpus were tested in random order, leading to a total of 200 trials. As for NU6, the keywords from each of the lists were presented in random order. The experiment was conducted in a single session of 1.5 to 2 hours in duration.

## Results

### Intersubject Variability

**Figure 3.**
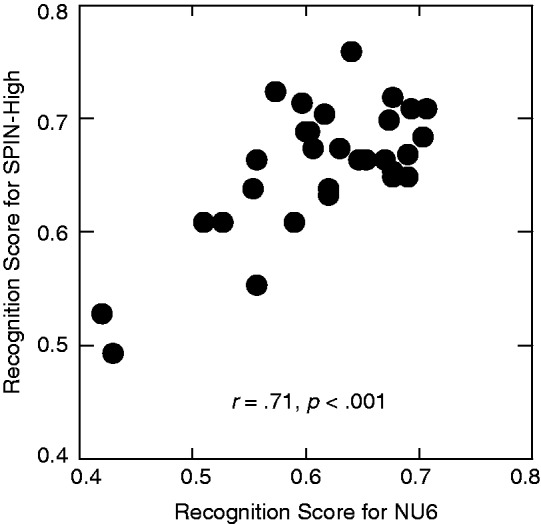
The scatterplot of the overall recognition scores from the qBIF procedure using the NU6 and SPIN-High test materials.

For the majority of the listeners, the percentage correct in recognizing the keywords was between 60% and 70% for both types of speech materials (see Figure 3). The one-step-ahead search algorithm of the qBIF procedure selected stimulus parameters that associated with 50% correct; therefore, the deviation from 50% observed here was mainly due to the recognition performance during the collection of the training data set at the beginning of the qBIF procedure. The overall recognition scores were correlated between the two test materials (*r* = .71, *p* < .001). This significant correlation suggests that the initial test condition of the qBIF procedure (with a TMR of 15 dB and one frequency band omitted) tends to be of different difficulties for different listeners. Moreover, the overall recognition score was higher for SPIN-High (65.9%) than NU6 (61.4%), *t*(29) = 4.55, *p* < .001. This difference in the overall score is dominated by the initial training portion of the qBIF procedure, *t*(29) = 5.38, *p* < .001, but not the trials after the stimulus optimization algorithm is activated, *t*(29) = 1.50, *p* = .144. This indicates that the initial test condition is associated with higher performance level for SPIN-High.

**Figure 4.**
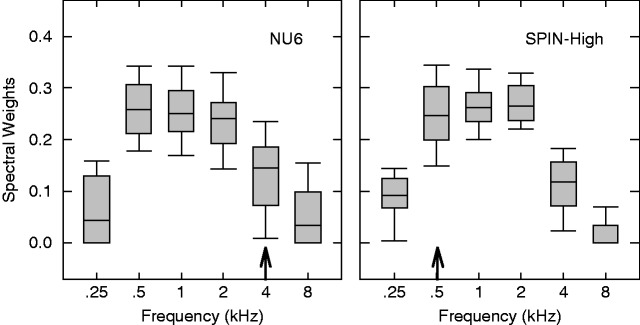
The band importance functions estimated using the qBIF procedure for the NU6 (left panel) and SPIN-High (right panel) materials. In each panel, the distributions of the estimated spectral weights across all listeners are plotted as a function of frequency bands. The distributions are represented using a box plot with the horizontal line within each box representing the median, the lower and upper box boundaries representing the 25th and 75th percentiles, and the lower and upper whiskers representing the 10th and 90th percentiles. The frequency band that exhibits the largest variances in spectral weights is marked with an arrow along the x-axis.

Figure 4 plots the spectral weights as functions of frequency bands (i.e., the band importance function) for the NU6 (left panel) and SPIN-High (right panel) materials. The overall shape of the band importance function was similar for both types of materials. The median spectral weights were the lowest in the 0.25-kHz and 8-kHz frequency bands, while the 0.5 -, 1 -, and 2-kHz bands exhibited the higher median weights.

Considerable individual differences in the spectral weights were observed. The range of the estimated weights across listeners was as much as 0.43 (NU6, 1 kHz), and the standard deviation, averaged across the six bands, was 0.07 for NU6 and 0.05 for SPIN-High. The frequency bands that exhibited the greatest variability were the 4-kHz band for NU6 (with a standard deviation of 0.078) and the 0.5-kHz band for SPIN-High (with a standard deviation of 0.072). These two bands are marked with arrows in Figure 3, and the spectral weights in these bands will be used to quantify the individual differences in the band importance functions.

Besides the band importance function, the SRT and the slope parameter *β* of the link function (as in Equation (4)) were also estimated. The average SRTs were −1.80 dB for NU6 and −2.70 dB for SPIN-High. The average *β* estimates were 0.15 (i.e., 3.85%/dB) for NU6 and 0.29 (i.e., 7.34%/dB) for SPIN-High. Therefore, the NU6 material led to higher SRTs, *t*(29) = 2.90, *p* = .007, and shallower link-function slopes, *t*(29) = 4.96, *p* < .001. For both types of speech materials, the estimated SRT and *β* were highly correlated, *r* = −.66, *p* < .001 for NU6 and *r* = −.88, *p* < .001 for SPIN-High. That is, lower SRTs are associated with steeper link-function slopes. For simplicity, the SRT will be used to quantify the individual differences in the link function.

**Figure 5.**
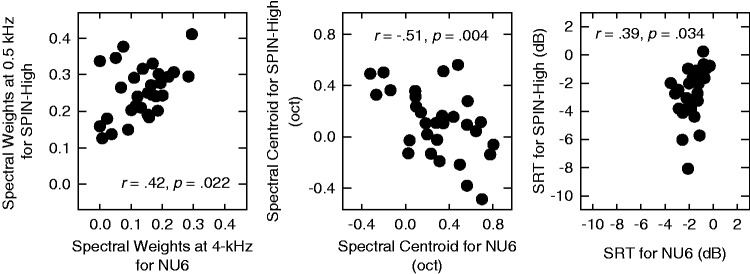
Left: the spectral weights at 0.5-kHz for SPIN-High against those at 4-kHz for NU6 as a scatterplot. Middle: the spectral centroids of the band importance functions for SPIN-High against those for NU6 as a scatterplot. Right: the SRTs for SPIN-High against those for NU6 as a scatterplot. In each panel, the correlation coefficient and the corresponding significance level are provided as inserted text. SRT = speech recognition threshold.

The key hypothesis of the current study is that the individual differences in the estimated parameters of the SII models are not random but consistent across measurements under different test conditions. Here, the individual differences are quantified using three metrics: (a) the spectral weights in the band with greatest variability (arrows in Figure 4), (b) the spectral centroid of the band importance function, and (c) the SRT. The spectral centroid is included to capture the shifts in the high-weight region in the band importance function. It is calculated as the average deviation from the center frequencies of the six bands to 1 kHz (in octaves), weighted by the spectral weights. If the hypothesis holds, then these metrics of individual differences should correlate across the two tests using two different speech materials. The correlations across the two test materials for the three metrics are represented in the three panels of Figure 5 as scatterplots.

Significant correlations were found for all three metrics of individual differences (*r* = .42, *p* = .022 for the spectral weights, *r* = −.51, *p* = .004 for the spectral centroid, and *r* = .39, *p* = .034 for the SRT). This means that at least a portion of the variability in the parameter estimates is based on the inherent characteristics of the listeners and these inherent characteristics tend to maintain themselves across different test conditions. Therefore, fitting the SII model to individual listeners using the qBIF procedure may improve the prediction of speech-recognition performance from individual listeners.

### The Effect of Different Speech Materials

Both types of speech materials used in the current study contained monosyllabic keywords. The main difference between them was in that the SPIN-High material provided semantic context, which could potentially facilitate the recognition of the keywords and change the way listeners weigh information across the spectrum.

**Figure 6.**
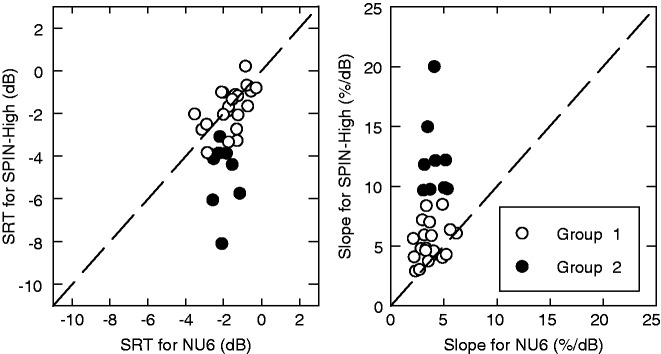
The SRT (left panel) and the link-function slope (right panel) estimates for SPIN-High against those for NU6 as scatterplots. In each panel, the listeners in the two groups identified by *k*-means clustering are indicated using filled (for Group 1) and unfilled (for Group 2) symbols. The diagonal dashed line indicates the line of equivalence. SRT = speech recognition threshold.

Although correlations were found between the parameter estimates from the measurements using the two speech materials, there were some noticeable material-dependent effects. For example, as described earlier, the SPIN-High materials corresponded to lower SRTs and steeper link-function slopes than NU6. Close inspections to individual data (e.g., see the right panel of Figure 5), however, suggests that not all listeners followed the trend of the average SRT and *β*. A *k-means* cluster analysis was performed based on the SRT and *β* estimates using both materials, which identified two listener groups. The estimates of the SRT and link-function slope are plotted in Figure 6 separately for the two listener groups. Listeners in Group 1 (unfilled symbols) had SRT and slope estimates that were very close for NU6 and SPIN-High (close to the diagonal dashed line), while listeners in Group 2 (filled symbols) had lower SRTs and steeper link-function slopes for SPIN-High compared with NU6. Therefore, the differences in the average SRT and slope estimates were mainly driven by Group 2.

To investigate the effect of speech material on the band importance function, a repeated measures analysis of variance was conducted, treating speech material and frequency bands as the two independent variables and spectral weight as the dependent variable. The spectral weights in the 0.25 - and 8-kHz bands were omitted from the analysis because they were very close to zero and not normally distributed. A significant main effect of frequency was found, *F*(3, 87) = 47.48, *p* < .001. The effect of speech material was not significant, *F*(1, 29) = 0.29, *p* = .592, neither was there a significant interaction between frequency and speech material, *F*(3, 87) = 1.87, *p* = .140. The lack of a significant interaction may be due to the poor spectral resolution of the qBIF procedure. The octave-frequency bands implemented in the qBIF procedure may be too coarse to reveal the effects of speech material on the shape of the band importance function.

As shown in the middle panel of Figure 5, the spectral centroids for the band importance functions are negatively correlated between the NU6 and SPIN-High materials. Moreover, this negative correlation was observed for both groups of listeners identified by the cluster analysis, (*r* = −.46, *p* = .035 for Group 1, and *r* = −.81, *p* = .001 for Group 2). This could be interpreted as that listeners who shift their spectral weights to higher frequencies for one material shift the spectral weights to lower frequencies for the other material. It is not clear whether these shifts in spectral weights are caused by contextual cues, since other differences between the two speech materials (e.g., acoustic differences between the two materials) could also contribute to such shifts. The current result warrants further systematic investigations on the effects of different speech materials.

## Discussion

### Comparison to Previous Studies

**Figure 7.**
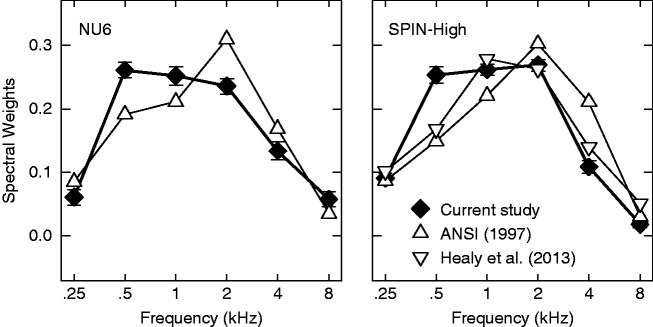
The average band importance functions estimated using the qBIF procedure in the current study (filled diamonds) for the NU6 (left panel) and SPIN-High (right panel) speech materials. Error bars indicate ± 1 standard error of the mean. The band importance functions provided by the ANSI standard (ANSI, 1997) are plotted using the upward triangles. The band importance function reported by Healy et al. (2013) for the SPIN-High materials (not the combined function of the SPIN-High and SPIN-Low materials) is plotted as downward triangles in the right panel.

The band importance function is an essential component of the SII model and it has been reported for various speech materials in the corresponding ANSI standard (1997). Figure 7 plots the average band importance function estimated in the current study (filled diamonds) with the band importance functions from the standard (upward triangles) for the NU6 (left panel) and SPIN-High (right panel) materials. For both materials, the band importance functions from the standard had lower spectral weights at 0.5 and 1 kHz and higher weights at 2 and 4 kHz.

The band importance function reported by Healy et al. (2013) for the SPIN-High material is also plotted in the right panel of Figure 7 (downward triangles). Healy et al. (2013) obtained their band importance function in 21 frequency bands for the SPIN-High material. To enable direct visual comparisons, the spectral weights for octave bands were derived from the 21-band data through summing spectral weights within an octave. Although the band importance function obtained by Healy et al. (2013) for the SPIN-High material exhibits a similar general shape as those in the standard and the current study, it has a lower weight at 0.5 kHz than the qBIF estimate and a lower weight at 4 kHz than the ANSI standard.

Differences in methodology are known to influence the estimates of the band importance function. The band importance functions in the ANSI standard were estimated by systematically low-pass and high-pass filtering the speech stimuli. As pointed out by a number of previous studies (e.g., Healy et al., 2013; Humes & Kidd, 2016; Warren, Bashford, & Lenz, 2005), this classic approach depended on the assumption that individual frequency bands contribute to speech intelligibility independently, but this assumption is frequently violated because adjacent frequency bands often share redundant speech information. As a work-around of this problem, the compound technique (e.g., Apoux & Healy, 2012) randomly presents a subset of the frequency bands without requiring the presented bands to be contiguous. Healy et al. (2013) have shown that the band importance functions estimated using the compound technique exhibited some noticeable differences from those in the ANSI standard. Most notably, for phonetically balanced words (CID-W22, Hirsh et al., 1952), large differences in spectral weights were observed across most of the frequency bands below 2 kHz.

Similar to the compound technique (Apoux & Healy, 2007; Healy et al., 2013), the qBIF procedure does not require the presented frequency bands to be adjacent to one another. On the other hand, the classic low-pass or high-pass approach (French & Steinberg, 1947; Studebaker et al., 1987) contributed to the data in the ANSI standard (1997) only presents adjacent bands. Therefore, it is expected that the band importance functions collected using the qBIF procedure are more akin to those collected using the compound technique than the low-pass or high-pass, classic approach. For each listener, the RMS deviations relative to the Healy et al. (2013) data and ANSI data were computed for the SPIN-High material. A paired *t*-test showed that the RMS deviation was smaller for the Healy et al. data than the ANSI data, *t*(29) = 5.86, *p* < .001. This suggests that the band importance function estimated in the current study for SPIN-High was closer to the corresponding band importance function reported by Healy et al. (with an average RMS deviation of 0.059) compared with the ANSI standard (with an average RMS deviation of 0.076).

### The Limitations of the qBIF Procedure

The qBIF procedure presented in the current study enables the estimation of the band importance function and the parameters of the SII model from individual listeners. This means that the SII model can be used to not only account for stimulus-originated effects, such as the effects of different speech materials, but also listener-originated effects, which provides a tool for modeling individual differences in speech recognition. On the other hand, the qBIF procedure does have a few limitations.

First, to gain computational efficiency and stability, the implementation of the SII model in the qBIF procedure has been kept very simple. In particular, the octave-frequency bands are used, which is quite coarse compared with the one-third-octave band importance functions available in the ANSI standard. Using the compound technique, Healy et al. (2013) showed that when the band importance functions are estimated at high resolution, they exhibit fine structures that could potentially improve the predictive power of the SII model. The octave bands adopted in the qBIF procedure are insufficient in capturing the fine spectral details in the band importance functions.

Second, due to the use of logistic regression in the qBIF procedure, the link function has been modeled as a logistic function (Equation (4)). Although this formulation is common for describing speech recognition performance (e.g., Brand and Kollmeier, 2002; MacPherson & Akeroyd, 2014), this is different from the SII model implemented in many previous studies. Traditionally, the link function in the SII model is frequently described by Equation (7). This formulation of the link function and its similar varieties (e.g., Boothroyd, 2008) are different from the logistic function, in that the function specified in Equation (7) is asymmetric for high and low SII values. That is, the slope of the link function for the formulation given in Equation (7) is asymmetric about the SRT, while for the logistic link function, the slope is symmetric about the SRT. The asymmetric link function agrees very well with behavioral results, potentially because it can partially capture the contextual effect in speech recognition (e.g., Boothroyd & Nittrouer, 1988). Therefore, it is possible that the performance of the qBIF procedure can be improved by adopting a generalized linear regression that uses an asymmetric link function rather than using a logistic function.

Finally, although the qBIF procedure is an adaptive procedure that determines the stimulus iteratively based on the previously collected responses, it is not completely free of a priori knowledge about the speech material or the listener. This is mainly because an initial training data set needs to be collected first. To ensure that this initial training set is sufficiently informative, the experimenter needs to select the initial TMR carefully. An initial TMR that is too high (i.e., the speech recognition is too easy) or too low (i.e., the speech recognition is too difficult) would lead to ceiling or floor performance for the training data, respectively. Consequently, the logistic regression would be ill-posed once the one-step-ahead search algorithm is activated, which would severely undermine the efficiency of the qBIF procedure. Therefore, the current implementation of the qBIF procedure requires the experimenter to have a rough estimate of the expected performance range for the speech material and listener. Although the selection of the initial TMR can be supported by previously reported results in the literature, the procedure to collect the initial training data may be modified in the future to reduce the amount of a priori knowledge required so that the qBIF procedure can be more easily applied to a wide range of speech materials and clinical populations.

## Summary

A Bayesian adaptive procedure, the qBIF procedure, was presented and utilized to fit the SII model to individual listeners’ speech recognition performance. Octave-frequency band importance functions for monosyllabic words (NU6) and monosyllabic words in high context sentences (SPIN-High) were estimated for each individual listeners. The spectral weights in the frequency bands that exhibited the greatest intersubject variability, the spectral centroids of the band importance functions, as well as the estimated SRTs were correlated between the two test materials. These results suggest that the variability in the estimated parameters of the SII model not only reflects stimulus differences but also differences inherent to individual listeners. Therefore, the SII framework can be useful in capturing individual differences in speech recognition.
